# Apple cider vinegar soaks do not alter the skin bacterial microbiome in atopic dermatitis

**DOI:** 10.1371/journal.pone.0252272

**Published:** 2021-06-02

**Authors:** Lydia A. Luu, Richard H. Flowers, Yingnan Gao, Martin Wu, Sofia Gasperino, Ann L. Kellams, DeVon C. Preston, Barrett J. Zlotoff, Julia A. Wisniewski, Steven L. Zeichner

**Affiliations:** 1 Department of Dermatology, University of Virginia, Charlottesville, Virginia, United States of America; 2 Department of Biology, University of Virginia, Charlottesville, Virginia, United States of America; 3 Division of Pediatric Infectious Diseases, Department of Pediatrics, Pendleton Pediatric Infectious Disease Laboratory, Child Health Research Center, University of Virginia, Charlottesville, Virginia, United States of America; 4 Division of General Pediatrics, Department of Pediatrics, University of Virginia, Charlottesville, Virginia, United States of America; 5 Division of Allergy and Immunology, Department of Pediatrics and Internal Medicine, University of Virginia, Charlottesville, Virginia, United States of America; Skin Research Institute Singapore, SINGAPORE

## Abstract

**Introduction:**

Atopic dermatitis is a common skin disease characterized by altered cutaneous immunity in which patients often exhibit lower skin microbiota diversity compared to healthy skin and are prone to colonization by *Staphylococcus aureus*. Apple cider vinegar has been shown to have antibacterial effects; however, its effects on the skin microbiome have not previously been well-described.

**Objectives:**

We aimed to examine the effects of topical dilute apple cider vinegar soaks on *Staphylococcus aureus* abundance, skin bacterial microbiome composition, and skin bacterial microbiome diversity in atopic dermatitis participants compared to healthy skin.

**Methods:**

Eleven subjects with atopic dermatitis and 11 healthy controls were enrolled in this randomized, non-blinded, single-institution, split-arm pilot study. Subjects soaked one forearm in dilute apple cider vinegar (0.5% acetic acid) and the other forearm in tap water for 10 minutes daily. Skin bacteria samples were collected from subjects’ volar forearms before and after 14 days of treatment. 16S sequencing was used to analyze *Staphylococcus aureus* abundance and skin bacterial microbiome composition, and alpha diversity of microbiota were determined using Shannon diversity index.

**Results:**

There was no difference in skin bacterial microbiome in atopic dermatitis subjects after 2 weeks of daily water or apple cider vinegar treatments (p = 0.056 and p = 0.22, respectively), or in mean abundance of *S*. *aureus* on apple cider vinegar-treated forearms (p = 0.60). At 2 weeks, the skin bacterial microbiomes of healthy control subjects were not significantly different from the skin bacterial microbiome of atopic dermatitis subjects (p = 0.14, 0.21, 0.12, and 0.05).

**Conclusions:**

Our results suggest that daily soaks in 0.5% apple cider vinegar are not an effective method of altering the skin bacterial microbiome in atopic dermatitis. Further studies are needed to explore the effects of different concentrations of apple cider vinegar on skin microflora and disease severity.

**Trial number:**

UVA IRB-HSR #19906.

## Introduction

Atopic dermatitis (AD), a common skin disease, affects up to 20% of children and 6% of adults [1]. AD is associated with significant utilization of health care resources, as patients with AD cost the health system $3,302 more annually in the United States than patients without AD [2]. AD is characterized by altered cutaneous immunity and skin barrier defects that increase susceptibility to bacterial infections [3, 4]. AD patients exhibit lower skin microbiota diversity compared to healthy skin [5], and over 90% of AD patients have colonization of lesional skin with *Staphylococcus aureus (S*. *aureus)* [6, 7], characteristics that have been targeted by AD treatments such as topical steroids [8]. Low density *S*. *aureus* is also present in non-lesional skin of AD patients and demonstrates resistance to common antimicrobials [9, 10]. In contrast, *S*. *aureus* is absent from the healthy skin microbiome, except in moist higher pH intertriginous zones and nares [11–13]. *S*. *aureus-*colonized patients have higher total serum IgE levels and a higher food allergy prevalence [14]. AD subjects with IgE directed towards staphyloccocal enterotoxins also have a higher incidence of asthma [15, 16].

Microbial dysbiosis and *S*. *aureus* colonization are driven by impairment of epidermal acidification in AD [11, 17–19]. Breakdown products of filaggrin, a key epidermal differentiation complex protein deficient in AD skin, contribute to epidermal acidification and impair *S*. *aureus* growth by forming pyrrolidone carboxylic acid and trans-urocanic acid (t-UCA) [20, 21]. The alkaline pH of AD skin likely arises from insufficient filaggrin-derived t-UCA; other natural acidifiers may contribute to skin flora dysbiosis [11, 22, 23].

AD is difficult to treat and current treatments are not curative. Bath additives like dilute sodium hypochlorite (bleach) are often recommended by dermatologists as adjuvant therapy to reduce disease severity due to their potential anti-staphylococcal benefits [24]. However, evidence supporting their effectiveness is sparse [13, 25–27]. Given the acid mantle impairment in atopic dermatitis, bleach is a counterintuitive approach from a pH standpoint to manage *S*. *aureus* [11, 22, 23]. In addition, dilute bleach neither improves skin pH nor eradicates *S*. *aureus* from AD skin [25, 28, 29]. Dilute bleach’s beneficial effects may be comparable to water baths alone [29]. In *ex-vivo* studies, bleach concentrations of greater than 0.03% sodium hypochlorite were required to eradicate *S*. *aureus* biofilms, but those levels are cytotoxic to human cells and should not be used clinically [30, 31]. Evidence-based alternatives to bleach that mitigate *S*. *aureus* are desirable.

There is increasing interest in complementary and alternative treatments for AD, especially apple cider vinegar given its antimicrobial properties. Dilute vinegar (AA range 0.16% to 0.31%) inhibits *ex-vivo* growth and biofilm formation of various human skin pathogens, including *S*. *aureus* [32]. ACV’s therapeutic potential for AD specifically is supported by murine models that, after treatment with topical vinegar cream (pH 3.5), showed lower eczema scores, increased stratum corneum hydration, and decreased transepidermal water loss, compared mice treated with vehicle alone (pH 5.5) [33].

However, in spite of widespread recommendation of dilute ACV baths by dermatologists, there is little high-quality data supporting its use for atopic dermatitis (34). One small case series showed that vinegar baths with topical treatment improved AD disease severity [34]. In contrast, a recent small study found that dilute ACV compresses did not reduce eczematous skin *S*. *aureus* burden [35]. Similarly, in our pilot study of 11 AD patients and 11 healthy controls, we showed that dilute ACV soaks did not improve skin barrier integrity as measured by transepidermal water loss and skin pH, and caused skin irritation in a majority of subjects [36]. In spite of theoretical and *ex-vivo* benefits, the effect of dilute ACV baths on *S*. *aureus* colonization and the skin microbiome are currently unknown [36].

In this study, we examined the effects of topical dilute ACV soaks on *Staphylococcus aureus* abundance, skin bacterial microbiome composition, and skin bacterial microbiome diversity in AD and healthy skin.

## Methods

### Study participants

**Fig 1:**
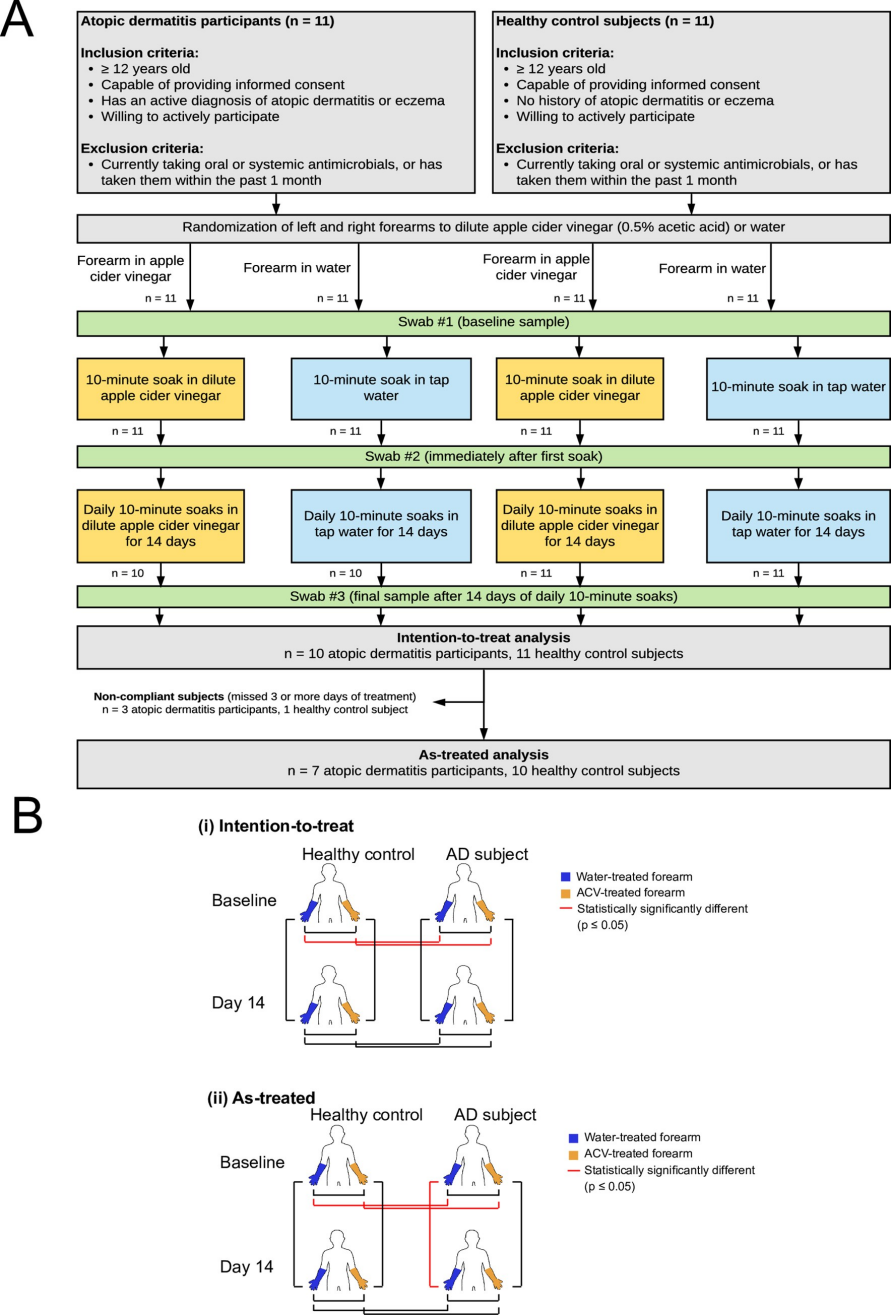
Study design. A, Study schematic. B, Diagrammatic representation of skin bacterial microbiome comparisons between forearms, (i) intention-to-treat and (ii) as-treated.

Participants with AD and healthy controls, age ≥ 12 years, were recruited through advertisement and from University of Virginia dermatology clinics [36]. Subjects were enrolled between June 2017 and October 2017 (**Fig 1A**). All subjects met U.K. AD diagnostic criteria [37]. Healthy controls had no current or prior history of AD. Subjects were excluded if they used topical or systemic antimicrobials within 1 month of enrollment. All other treatments used by subjects for AD, including topical steroids and immune modulators, were recorded. Subjects were asked to continue these treatments as prescribed during study participation. All participants or guardians signed written informed consent; minors provided verbal assent. The University of Virginia Institutional Review Board approved the study.

### Intervention

Patient characteristics were ascertained by questionnaire and medical record review. Disease severity was determined using the Severity Scoring of Atopic Dermatitis Index (SCORAD) [38]. Following a standardized education session, AD and control subjects followed identical written protocols for soaking one forearm in dilute ACV (0.5% concentration acetic acid) and the other in tap water for 10 minutes daily [36]. Participants were taught to prepare soaks (below) and supervised soaking their forearms at the initial visit, then instructed to follow the procedure every day for 2 weeks. The forearm selected for ACV treatment was determined by a pseudo-random number generator before recruitment.

### Preparation of apple cider vinegar and tap water soaks

White House Foods® Apple Cider Vinegar and 3-gallon soaking basins were provided to all subjects. ACV was diluted to 0.5% AA by mixing 2.4 cups of ACV with 21.6 cups of tap water in a 3-gallon soaking basin. A concentration of 0.5% AA was selected based on previous data regarding safety, tolerability, and antimicrobial qualities [39]. A second basin was used to soak the opposite forearm in tap water simultaneously. Subjects were instructed to rinse basins with tap water between uses.

### Data collection

Samples for analysis of skin microbiota were collected at study visit 1 before and after the first soak, and at study visit 2 after 14 days of daily soaking. For study visit 1, ACV and water soaks were performed on opposite forearms simultaneously. Skin bacteria samples were collected from subjects’ forearms using e-Swab^TM^ Liquid Amies Collection and Transport System (Copan e-Swab, Brescia, Italy). Swabs were rubbed across the volar forearm skin in a circular motion for 15 seconds, then placed into the transport system. Skin bacteria samples from study visit 1 before soaking and study visit 2 were analyzed.

Total DNA was isolated using modifications of previously described procedures, lysing samples with a lysozyme/mutanolysin/lysostaphin cocktail, followed by proteinase K/SDS treatment, and bead beating with a FastPrep-24 instrument [40, 41]. DNA was further purified with a Quick-DNA Fecal/Soil Microbe Kit (Zymo Research). DNA quality was assessed spectrophotometrically and with gel electrophoresis. Negative extraction controls were processed in parallel with each extraction.

16S amplicon sequencing was performed using V1-V3 primers on DNA extracted from swabs at baseline and at 2 weeks. The V1-V3 hypervariable regions of 16S rRNA were amplified through PCR with broad range primers 27F and 534R [42] (PCR amplification: New England Biolabs Phusion High-Fidelity PCR Master Mix with HF Buffer: M0531S; PCR primers: custom primers ordered from Integrated DNA Technologies; reaction purification/normalization: Applied Biosystems SequalPrep™ Normalization Plate Kit, 96-well: A1051001). 16S rRNA amplicon libraries were pooled and sequenced using MiSeq Reagent Kit v3 (Illumina MiSeq Reagent Kit v3 (600-cycle): MS-102-3003) and the Illumina MiSeq instrument. Microbial amplicon variants present in each sample were identified and their relative abundances were quantitated using DADA2 for quality filtering, chimeric sequence removal, identification of unique amplicon variants, and taxonomic classification [43]. Sequences read were submitted to the National Center for Biotechnology Information, US National Library of Medicine, NIH, Sequence Read Archive under accession number PRJNA639330 and are freely available.

### Statistical analysis

We calculated the Aitchison distance [44] using R package ‘microbiome’ to quantify differences between skin microbiota compositions [45]. We applied permutational multivariate analysis of variance (PERMANOVA) using R package ‘vegan’ to partition variance of Aitchison distance between fixed effects (i.e., status of AD, time after treatment and type of treatment) and random effects. By examining R-squared and the p-values of each condition or treatment, we determined if that condition or treatment results in significant microbiota compositional change. Statistical power of PERMANOVA was determined using R package ‘micropower’ [46]. Random forest analysis was applied using R package ‘randomForest’ to identify genera that distinguish microbiota from the AD subjects and controls at baseline [47]. Shannon diversity index was used to quantify skin microbiota diversity through R package ‘microbiome’. We analyzed data on both intent-to-treat and as-treated bases. For intent-to-treat analysis, all subjects were included in the analysis, regardless of treatment adherence. For as-treated analysis, subjects who missed three or more days of treatment were excluded.

## Results

### Characteristics of AD and control subjects

Eleven subjects with AD (geometric mean (GM) age 20.6 years) and 11 healthy subjects (GM age 28.8 years) were enrolled. **Table 1** summarizes baseline characteristics of AD and healthy subjects. The majority of AD subjects had mild-to-moderate disease (GM SCORAD 32.9 [23.8–45.4]). Emollients and topical corticosteroids were the most commonly used treatments. Atopic dermatitis was present on volar forearms in 8/11 (73%) of AD subjects.

**Table 1:** Characteristics of study participants at enrollment.

		Atopic Dermatitis N = 11	Control Subjects N = 11	p-value
**Age (years)**^**1**^		20.6 [16.2–26.2]	28.8 [22.6–36.8]	0.15
**Gender**^**2**^	Male	36%	45%	1.00
	Female	64%	55%	
**Ethnicity**^**2**^	Caucasian	46%	36%	1.00
	African American	18%	0%	
	Asian	36%	46%	
	Other	0%	18%	
**Birth history**^**2**^	Vaginal delivery	73%	91%	0.59
	Caesarian delivery	27%	9%	
**Medical history**^**2**^	Allergies^3^	100%	36%	0.0039
**Family history**^**2**^	Allergies^3^	100%	64%	0.09
**SCORAD**^**1**^		33 [23.8–45.4]	0 [0–0]	N/A
**AD severity**^**4**^	Mild	36%	N/A	<0.01
	Moderate	46%		
	Severe	18%		
**AD medications**^**2**^	Emollients	100%	0%	<0.01
	Topical steroids	91%	0%	
	Systemics	18%	0%	
**Skin barrier**^**1**^	TEWL	11.1 [8.6–14.3]	7.1 [6.0–8.4]	0.0064
	pH	4.88 [4.67–5.1]	4.86 [4.6–5.13]	1.00
**Presence of dermatitis**^**2**^	ACV-treated forearm	73%	0%	<0.01
	Water-treated forearm	73%	0%	

^1^ Geometric mean [95% confidence interval]

^2^ Percentage of subjects (prevalence)

^3^ Positive history of allergies included asthma, food, or environmental allergies

^4^ AD severity was mild (if SCORAD <25), moderate (if SCORAD 25–50), or severe (if SCORAD >50).

### Effect of 14 daily 10-minute ACV soaks on skin microbiota

**Fig 2:**
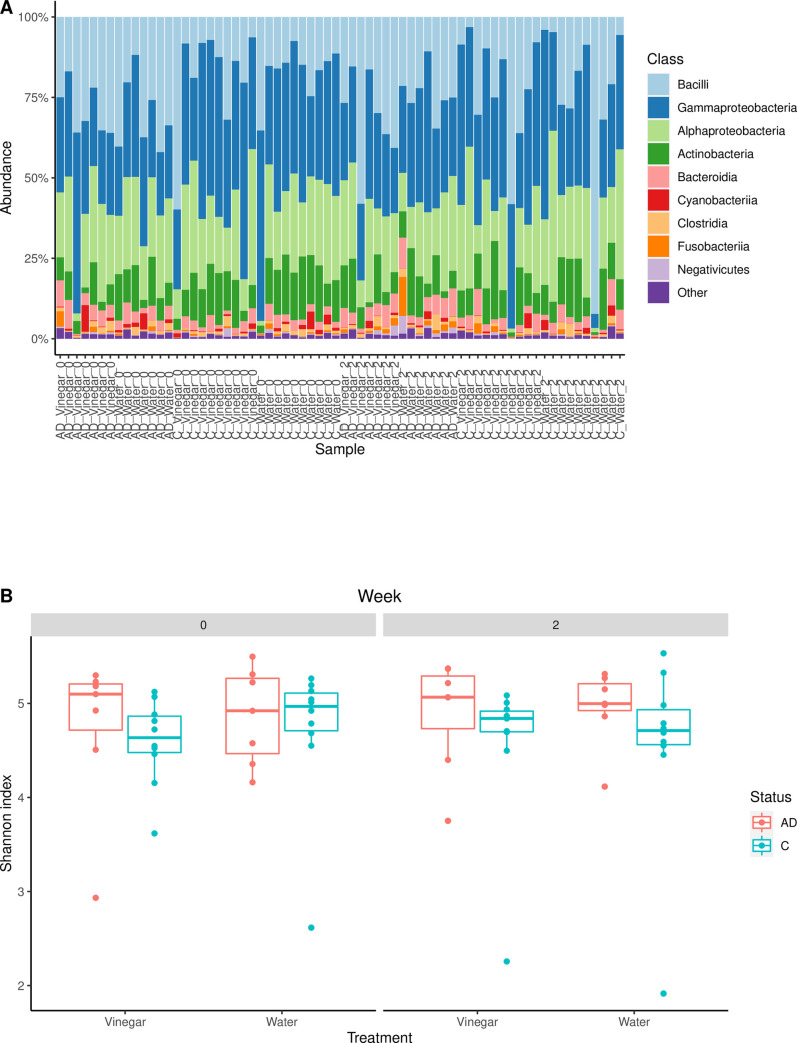
Skin microbiota composition and diversity. A, Stacked bar plots of bacterial taxonomic compositions in the skin microbiome, as-treated. B, Shannon diversity plot, as-treated.

At baseline, the bacterial microbiomes in AD participants and control subjects were significantly different (p = 0.011) (**Table 2, Figs 1B and 2A**). Disease status explained 6.2% of the variations in skin bacterial microbiome composition. Random forest analysis of baseline samples at the genus level revealed 8 genera that distinguished AD subjects and healthy controls: *Halomonas*, *Delftia*, *Massilia*, *Cutibacterium*, *Shewanella*, *Leuconostoc*, *Sphingomonas*, and *Staphylococcus*. These genera had an average abundance greater than 0.1% in at least one group. *S*. *aureus* was significantly more abundant on forearms of AD subjects compared to healthy controls (intention-to-treat analysis: 10.74% vs 0.01% respectively, p <0.001; as-treated analysis: 5.73% vs 0.01% respectively, p <0.001) (**Table 3**). There were also no significant differences in microbiota composition between the water-treated and ACV-treated forearms of healthy controls or AD subjects at baseline (intention-to-treat analysis: p = 0.468 for healthy controls, p = 0.385 for AD subjects; as-treated analysis: p = 0.731for healthy controls, p = 0.438 for AD subjects) (**Table 2, Figs 1B and 2A**).

**Table 2:** Pairwise PERMANOVA of skin microbiota composition.

**Intention-to-treat**			**Baseline**				**Day 14**			
			**Healthy control**		**AD subject**		**Healthy control**		**AD subject**	
			**Water-treated forearm**	**ACV-treated forearm**	**Water-treated forearm**	**ACV-treated forearm**	**Water-treated forearm**	**ACV-treated forearm**	**Water-treated forearm**	**ACV-treated forearm**
Baseline	Healthy control	Water-treated forearm		R^2^ = 0.0371	R^2^ = 0.0544	R^2^ = 0.0545				
				P = 0.4675	P = 0.0350	P = 0.0609				
				β = 0.662	β = 1.000	β = 0.991				
		ACV-treated forearm			R^2^ = 0.0596	R^2^ = 0.0615				
					P = 0.0200	P = 0.0110				
					β = 1.000	β = 0.999				
	AD subject	Water-treated forearm				R^2^ = 0.0314				
						P = 0.3846				
						β = 0.746				
		ACV-treated forearm								
Day 14	Healthy control	Water-treated forearm	R^2^ = 0.0384	R^2^ = 0.0418	R^2^ = 0.0507	R^2^ = 0.0497		R^2^ = 0.0335	R^2^ = 0.0525	R^2^ = 0.0529
			P = 0.6623	P = 0.0969	P = 0.1499	P = 0.2298		P = 0.5055	P = 0.1818	P = 0.1538
			β = 0.571	β = 0.890	β = 0.999	β = 0.992		β = 0.647	β = 0.995	β = 0.998
		ACV-treated forearm	R^2^ = 0.0387	R^2^ = 0.0390	R^2^ = 0.0528	R^2^ = 0.0526			R^2^ = 0.0518	R^2^ = 0.0551
			P = 0.7283	P = 0.4685	P = 0.0879	P = 0.1089			P = 0.2348	P = 0.1059
			β = 0.526	β = 0.669	β = 1.000	β = 0.998			β = 0.999	β = 1.000
	AD subject	Water-treated forearm	R^2^ = 0.0534	R^2^ = 0.0563	R^2^ = 0.0384	R^2^ = 0.0401				R^2^ = 0.0339
			P = 0.1179	P = 0.0599	P = 0.2947	P = 0.3506				P = 0.3227
			β = 0.998	β = 1.000	β = 0.570	β = 0.606				β = 0.602
		ACV-treated forearm	R^2^ = 0.0570	R^2^ = 0.0627	R^2^ = 0.0371	R^2^ = 0.0378				
			P = 0.0340	P = 0.0130	P = 0.7822	P = 0.6014				
			β = 1.000	β = 1.000	β = 0.323	β = 0.557				
**As-treated**			**Baseline**				**Day 14**			
			**Healthy control**		**AD subject**		**Healthy control**		**AD subject**	
			**Water-treated forearm**	**ACV-treated forearm**	**Water-treated forearm**	**ACV-treated forearm**	**Water-treated forearm**	**ACV-treated forearm**	**Water-treated forearm**	**ACV-treated forearm**
Baseline	Healthy control	Water-treated forearm		R^2^ = 0.0417	R^2^ = 0.0733	R^2^ = 0.0699				
				P = 0.7313	P = 0.0140	P = 0.0939				
				β = 0.508	β = 1.000	β = 0.957				
		ACV-treated forearm			R^2^ = 0.0845	R^2^ = 0.0823				
					P = 0.0070	P = 0.0170				
					β = 1.000	β = 0.999				
	AD subject	Water-treated forearm				R^2^ = 0.0519				
						P = 0.4375				
						β = 0.294				
		ACV-treated forearm								
Day 14	Healthy control	Water-treated forearm	R^2^ = 0.0458	R^2^ = 0.0486	R^2^ = 0.0697	R^2^ = 0.0661		R^2^ = 0.0390	R^2^ = 0.0698	R^2^ = 0.0676
			P = 0.4056	P = 0.0829	P = 0.0639	P = 0.2078		P = 0.4755	P = 0.0649	P = 0.1069
								β = 0.591	β = 0.995	β = 0.983
			β = 0.595	β = 0.820	β = 0.990	β = 0.953				
		ACV-treated forearm	R^2^ = 0.0446	R^2^ = 0.0455	R^2^ = 0.0740	R^2^ = 0.0699			R^2^ = 0.0681	R^2^ = 0.0688
			P = 0.6723	P = 0.3317	P = 0.0310	P = 0.0889			P = 0.1269	P = 0.1039
			β = 0.452	β = 0.657	β = 1.000	β = 0.974			β = 0.990	β = 0.984
	AD subject	Water-treated forearm	R^2^ = 0.0668	R^2^ = 0.0736	R^2^ = 0.0652	R^2^ = 0.0625				R^2^ = 0.0477
			P = 0.1299	P = 0.0230	P = 0.0312	P = 0.3281				P = 0.5938
			β = 0.988	β = 0.997	β = 0.851	β = 0.335				β = 0.172
		ACV-treated forearm	R^2^ = 0.0680	R^2^ = 0.0749	R^2^ = 0.0602	R^2^ = 0.0585				
			P = 0.0979	P = 0.0410	P = 0.2500	P = 0.2969				
			β = 0.984	β = 0.990	β = 0.339	β = 0.437				

Key:

Yellow: Statistically significantly different (p < 0.05).

Orange: Statistically significantly different (p < 0.05), but more than one factor has changed between the two groups being compared.

**Table 3:** Mean and median abundance of *Staphylococcus aureus* on ACV-treated forearms. The median abundances are shown in the parentheses.

	Intention-to-treat		As-treated	
	Healthy control	AD participant	Healthy control	AD participant
**Baseline**	0.01% (0.00%)	10.74% (1.16%) *	0.01% (0.00%)	5.73% (1.06%) *
**2 weeks**	0.05% (0.01%)	6.37% (0.81%) ^†^	0.05% (0.02%)	3.61% (0.59%) ^†^

* pairwise comparison vs healthy control at baseline, p < 0.05 by Wilcoxon rank-sum test.

† pairwise comparison vs healthy control at 2 weeks, p < 0.05 by Wilcoxon rank-sum test.

We then examined the effects of 2 weeks of daily ACV treatments. **Fig 1B** illustrates skin microbiota significant comparisons on both an intention-to-treat and as-treated basis. Analyses on an intention-to-treat basis showed no difference in skin bacterial microbiota in healthy control subjects after 2 weeks of daily water or ACV treatments (PERMANOVA, p = 0.662and p = 0.469, respectively) (**Table 2**), or in mean abundance of *S*. *aureus* on ACV-treated forearms (Wilcoxon signed rank test, p = 0.349). There was also no difference in skin bacterial microbiome in AD subjects after 2 weeks of daily water or ACV treatments (PERMANOVA, p = 0.295 and p = 0.601, respectively). Although the mean abundance of *S*. *aureus* on ACV-treated forearms seems to decrease after 2 weeks of daily ACV treatment, such decrease was mostly contributed by a few individuals with drastic drop of *S*. *aureus* abundance and was not consistent among individuals. The median abundance of *S*. *aureus* on ACV-treated arms showed less evident decrease, and the drop in the mean abundance was not significant (Wilcoxon signed rank test, p = 0.105). Interestingly, after 2 weeks of daily treatments, the skin bacterial microbiome of forearms treated with water were not significantly different from those treated with ACV in either healthy control subjects or AD subjects (PERMANOVA, p = 0.506 and p = 0.323, respectively). At 2 weeks, the skin bacterial microbiomes of healthy control subjects were not significantly different from the skin bacterial microbiome of AD subjects (PERMANOVA, p = 0.182, 0.235, 0.154, and 0.106) (**Table 2**), although mean abundance of *S*. *aureus* remained significantly higher on forearms of AD subjects (Wilcoxon rank sum test, p <0.001) (**Table 3**). When analyzed on an as-treated basis where non-compliant subjects were excluded, results were similar, except there was a significant change in skin bacterial microbiome of forearms of AD subjects that were treated with water daily for 2 weeks (p = 0.031) (**Table 2**). Power analysis using R package ‘micropower’ on as-treated data showed that the effect size of compositional change in microbiota before and after water or ACV treatment is very close to zero, resulting in a median power of 0.48 (ranging from 0.17 to 0.85) in PERMANOVA. To get a statistical power > 0.9 to detect compositional change before and after water or ACV treatment similar to what was observed in this study, at least 15 patients are required for each group. On the other hand, the effect size in microbiota between AD patients and healthy controls ranges from 0.003 to 0.022, yielding a power > 0.9 in PERMANOVA.

Kruskal-Wallis test by ranks of Shannon diversity indices revealed no significant differences in microbiota diversity of any forearms at any point in time (p = 0.474, **Fig 2B**).

## Discussion

We found AD subjects have different skin microbiota composition compared to healthy controls at baseline. In particular, *Staphylococcus* was more abundant in AD subjects, while *Halomonas*, *Delftia*, *Massilia*, *Cutibacterium*, *Shewanella*, *Leuconostoc*, *and Sphingomonas* were less abundant, findings consistent with the literature [4, 10]. However, although prior studies had found that healthy controls have higher skin microbiome diversity compared to AD subjects [5], we did not find any significant differences in skin bacterial microbiota diversity between AD subjects and healthy controls at baseline. As expected, at baseline there was no difference between skin bacterial microbiota of forearms to be treated with water and forearms to be treated with ACV in either healthy controls or AD subjects.

In both intent-to-treat and as-treated analysis, healthy control subjects showed no difference from baseline skin bacterial microbiome after 2 weeks of either daily water treatments or daily ACV treatments. This is consistent with prior studies showing that the skin microbiota exhibits temporal stability despite environmental disruptions in healthy adults [48, 49]. Therefore, 2 weeks of daily 10-minute treatments with dilute ACV was not sufficient to change the skin bacterial microbiome of healthy controls. However, we also found that the skin microbiota and mean abundance of *S*. *aureus* on forearms of AD subjects showed no significant changes after 14 days of daily ACV treatment, and the forearms treated with ACV were not significantly different from those treated with water. These results were surprising, as we had hypothesized that the AD skin bacterial microbiome would be more susceptible to changes due to environmental perturbations such as daily ACV treatments, and that ACV would have antimicrobial properties against *S*. *aureus*. The fact that we did not see a change shows that AD skin microbiomes may be more resilient to environmental perturbations than anticipated. Of note, previous studies have found that antiperspirant use significantly changes the axillary skin microbiome [50, 51] and cosmetic products affect the diversity of the facial skin microbiome [52]. Water treatments also did not produce significant differences in skin bacterial microbiome compared to dilute ACV in either controls or AD subjects, suggesting that water treatment was equivalent to dilute ACV treatment.

In spite of lack of significant changes in the skin bacterial microbiome after 2 weeks of ACV soaks in either healthy controls or AD subjects compared to baseline, we do note that after 2 weeks there were no longer any significant differences in skin bacterial microbiome between healthy controls and AD subjects, and no significant differences in skin bacterial microbiome diversity either. This finding suggests that although water and ACV treatments did not produce a statistically significant change in skin bacterial microbiome over the 2-week timespan, the treatments did cause a small enough change to cause the skin bacterial microbiomes of AD subjects to become more similar to the skin bacterial microbiomes of healthy controls. When analyzed on an as-treated basis, 2 weeks of daily water treatments caused a significant change in skin bacterial microbiome in AD subjects, and the skin bacterial microbiome in AD subjects became similar to that of healthy controls. This suggests that hydration of the skin is important in maintaining the microbiome of AD patients. This significance is lost when analyzing the data on an intention-to-treat basis, so the burden of treatment should be considered carefully. Not all patients will be able to adhere to a daily 10-minute soaking regimen; this may not be a valuable treatment option except in very motivated patients. Daily compresses that allow the subject to retain mobility during treatment time or relaxing full-body baths may be more acceptable options. It might be possible to observe significant effects if patients were treated more intensively or for longer times, but our study non-adherence rate of 3 of 11 AD subjects and 1 of 11 healthy controls missing 3 or more days of treatment over 14 days suggests that more intense interventions would only be possible for very highly motivated patients. It is also possible that our study was underpowered. However, given that the magnitude of the effects we observed were small, it would be reasonable to question the clinical significance of quantitatively small albeit statistically significant effects. A conservative interpretation of our observations would suggest that the effects of the ACV soak regimen used in our study are small in any case.

The concentration we chose of 0.5% AA is nearly 10-fold above the 1:80 (0.06% AA) ACV bath preparations recently recommended by Lee and Jacobs [53]. 0.5% AA is comparable to the MIC of 0.312% AA against methicillin-sensitive *S*. *aureus* (MSSA) and 0.625% AA against methicillin-resistant *S*. *aureus* (MRSA) [32]. While concentrations of 2.5% AA have been used in burn centers to prevent *P*. *aeruginosa* wound infections, concentrations above 3% AA have been associated with pain and itching [39]. In our study, *S*. *aureus* colonization persisted after 14 days of 10-minute soaks with 0.5% AA by PCR analysis, indicating that 0.5% AA from ACV has no lasting biocidal activity on AD skin already colonized with *S*. *aureus*. Although we did not test for MRSA in this study, the lack of efficacy of 0.5% AA was possibly due to subject colonization with resistant *S*. *aureus* strains.

This study has several limitations. First, this study examined a fairly small, homogenous group of patients, as the average ages of AD and control subjects were 20.6 and 28.8 years, respectively. Of note, the AD group of subjects included some participants of adolescent age, while the control group of subjects were all adults (over age 18). The skin microbiota shifts as people move from childhood to adulthood due to changing levels of sebum production, lipid content, pH, and hair growth, and so there are different microbial signatures associated with AD at different ages [54]. Therefore, this study may be less applicable to children. Further studies among a larger and more diverse group of patients are needed to further characterize the full effect of topical ACV treatments on skin microbiomes. Second, this study was unblinded due to the intrinsic appearance and odor of dilute ACV. Third, our study analyzed a single brand and dilution of ACV. Future studies should examine different concentrations of AA from different sources. Lastly, this study focused on the bacterial microbiome. It is well-known that other organisms, such as *Malessezia*, are important components of the skin microbiome that may also provoke inflammatory reactions and contribute to skin diseases such as atopic dermatitis [55]. Therefore, future studies should include a fungal ITS analysis as well.

In conclusion, apple cider vinegar is prominent among emerging natural remedies used in AD in spite of sparse evidence [53]. Our results show that even though daily soaks in 0.5% ACV do not change skin bacterial microbiomes significantly compared to water and are not likely a useful agent to affect skin-colonizing *S*. *aureus*, they may cause AD skin microbiome to become more similar to controls. Further studies are needed to explore whether ACV at different concentrations can promote a healthier skin microflora and modify disease severity.
